# A Non-Invasive Droplet Digital PCR (ddPCR) Assay to Detect Paternal *CFTR* Mutations in the Cell-Free Fetal DNA (cffDNA) of Three Pregnancies at Risk of Cystic Fibrosis via Compound Heterozygosity

**DOI:** 10.1371/journal.pone.0142729

**Published:** 2015-11-11

**Authors:** Emmanuel Debrand, Alexandra Lykoudi, Elizabeth Bradshaw, Stephanie K. Allen

**Affiliations:** 1 West Midlands Regional Genetics Laboratory, Birmingham Women's NHS Foundation Trust, Mindelsohn Way, Edgbaston, Birmingham, B15 2TG, United Kingdom; 2 Department of Medical Genetics, Athens University, Agia Sofia Children's Hospital, 11527, Athens, Greece; University of Bonn, Institut of experimental hematology and transfusion medicine, GERMANY

## Abstract

**Introduction:**

Non-invasive prenatal diagnosis (NIPD) makes use of cell-free fetal DNA (cffDNA) in the mother’s bloodstream as an alternative to invasive sampling methods such as amniocentesis or CVS, which carry a 0.5–1% risk of fetal loss. We describe a droplet digital PCR (ddPCR) assay designed to inform the testing options for couples whose offspring are at risk of suffering from cystic fibrosis via compound heterozygosity. By detecting the presence or absence of the paternal mutation in the cffDNA, it is possible to predict whether the fetus will be an unaffected carrier (absence) or whether further invasive testing is indicated (presence).

**Methods:**

We selected a family in which the parents were known to carry different mutated CFTR alleles as our test system. NIPD was performed for three of their pregnancies during the first trimester (at around 11–12 weeks of gestation). Taqman probes were designed against an amplicon in exon 11 of the CFTR gene, to quantify the proportion of mutant (ΔF508-MUT; FAM) and normal (ΔF508-NOR; VIC) alleles at position c.1521_1523 of the CFTR gene.

**Discussion:**

The assay correctly and unambiguously recognized the ΔF508-MUT CFTR allele in the cffDNA of all three proband fetuses and none of the six unaffected control fetuses. In conclusion, the Bio-Rad QX100 was found to be a cost-effective and technically undemanding platform for designing bespoke NIPD assays.

## Introduction

Non-invasive prenatal diagnosis (NIPD) is a rapidly-expanding technique in genetic diagnosis which exploits the presence of cell-free fetal DNA (cffDNA) circulating in the mother’s bloodstream, which is derived from the placental trophoblast. It can be carried out during the first trimester of pregnancy and avoids the complications of invasive testing by amniocentesis or chorionic villus sampling, which carry a small risk of miscarriage.

The principal challenge of NIPD is that cffDNA makes up only a small proportion of total free circulating DNA (generally less than 10% [1],[2]), especially at earlier stages of pregnancy, therefore high-sensitivity technology such as next-generation sequencing [3], microarrays [4] or quantitative PCR [5] is required to accurately detect it. Digital PCR (dPCR) is a highly sensitive and quantitative alternative to conventional qPCR which is based on the nanofluidic partitioning of DNA molecules between many small wells following a high degree of sample dilution. A positive reaction in a well indicates the presence of a single DNA molecule and the proportion of positive wells in the experiment is used to estimate the concentration of target molecules. See Zimmermann *et al*. (2008) [6], Lo and Chiu (2012) [7] or Barrett and Chitty (2014) [8] for reviews of the mechanism and its application to prenatal diagnosis. Droplet digital PCR (ddPCR) is a high-throughput nanofluidic version of this technique which uses around 20,000 wells.

Many dPCR/ddPCR-NIPD assays so far have been aimed at sex determination and aneuploidies (in particular Down syndrome) [2][9][10]. However it is also suitable for testing for polymorphic single-gene (Mendelian) disorders such as sickle cell disease [1] or β-thalassaemia [11] (*HBB* gene), or α(0)-thalassemia [12] (*SEA* allele), or in detecting rhesus-positive fetuses in alloimmunised pregnancies [13]. Some autosomal recessive diseases such as haemophilia [14] and cystic fibrosis (CF) are caused by many different alleles of the same gene, in which case it would be an advantage to use a platform which allows straightforward and economically-viable development of new assays on a “patient- or disease-specific basis [8]”. ddPCR is ideal in that the cost and level of expertise required to design and interpret a new assay are low.

Technology is currently progressing extremely rapidly in this area and we believe that it is crucial for genetics labs to share the results of evaluating new methodologies.

We report a proof-of-principle study in which a new Taqman-based ddPCR-NIPD assay was designed to determine the genotypes of fetuses of a couple carrying different mutated *CFTR* alleles.

## Materials and Methods

### Subjects and samples

**Fig 1:**
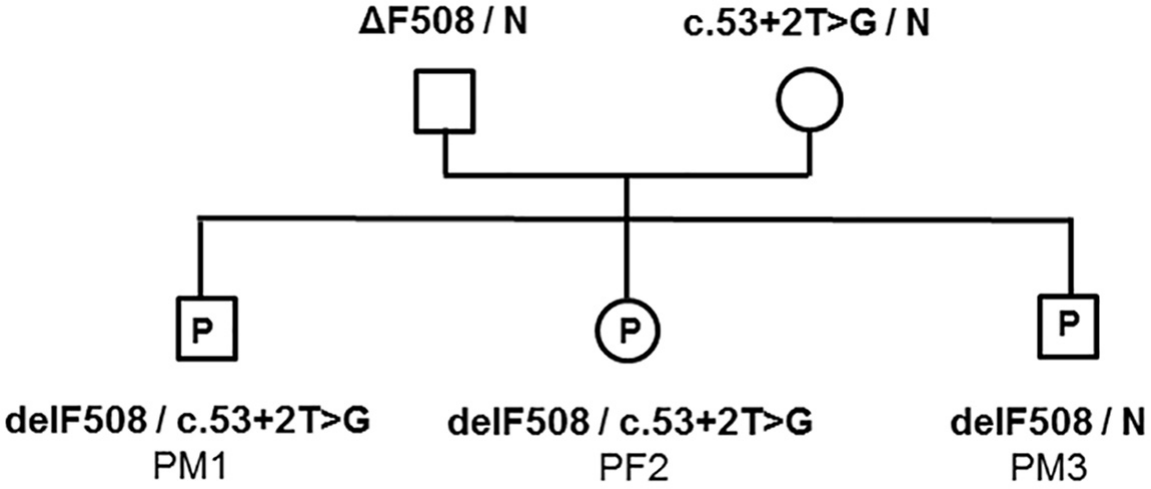
Pedigree of a family likely to benefit from non-invasive prenatal diagnosis. The parents each carried a different mutation, putting offspring at risk of compound heterozygosity. Three proband pregnancies (P) were tested, as well as three male and three female unrelated control pregnancies (not shown). Non-invasive testing was performed at around week 11–12 of gestation.

We selected a family in which the parents were known to carry different mutated CF alleles as our test system (Fig 1). The father carried the commonest mutation ΔF508 (c.1521_1523delCTT), while the mother carried the rarer mutation 185+2T>G (c.53+2T>G). Six normal unrelated pregnancies were used as controls (three male and three female fetuses). Maternal peripheral blood was sampled at around 11–12 weeks of gestation and DNA from the supernatant was extracted using the QIAamp Circulating Nucleic Acid Kit (Qiagen, Germany) according to the manufacturer’s instructions. Samples were named according to their sex and genotype: for example sample PM1 is a male proband, while sample CF2 is a female control.

### Assay design

The ZFX/ZFY assay to detect male cffDNA was used as previously described by Lun et al. [15]. New Taqman probes were designed for a CF-specific assay, against an amplicon in exon 11 of the *CFTR* gene, to quantify the proportion of mutant (ΔF508-MUT; FAM) and normal (ΔF508-NOR; VIC) alleles at position c.1521_1523 of the CFTR gene.

### DNA extraction, labelling and detection

Maternal venous blood was extracted during the first trimester. The plasma fraction was separated and stored at -80°C using standard procedures [1]. DNA was extracted using the QIAamp Circulating Nucleic Acid kit (Qiagen), according to the manufacturer’s instructions from 2 ml (samples PF2, CF4, CF5, CM1 and CM2), 3 ml (PM1, CF6 and CM3) or 4 ml (PM3) of plasma. DNA was eluted in 70 μL of AVE buffer. ZFX and ΔF508-NOR were labelled with VIC and detected at 535–555 nm. ZFY and ΔF508-MUT were labelled with FAM and detected at 510–530 nm.

### ddPCR

A general workflow for using the Bio-Rad QX100 system was recently described by Mazaika and Homsy [16]. All samples were analysed in triplicate (with the exception of sample CF6, which was analysed in duplicate) on the QX100 Droplet Digital PCR system (Bio-Rad, CA) according to the manufacturer’s instructions. 9 μl of DNA per 20 μl ddPCR reaction was used in each replicate. The sequence of primers and probes and PCR conditions are described in the supplementary materials (S1 Table). Further information about the PCR conditions is also provided in the supplementary materials (S1 Appendix). Male cffDNA quantification: a previously-described [17] ZFX/ZFY Taqman assay was used to detect and quantify the proportion of male fetal DNA (Pf) in all samples. CF allelic discrimination: a new duplex Taqman assay was designed to discriminate between the normal allele (ΔF508-NOR probe, dye = VIC-MGB) and the paternal mutant allele (ΔF508-MUT probe, dye = FAM-MGB) of exon 11 of the *CFTR* gene. The specificities of these probes were verified using anonymised genomic DNA from patients with and without the ΔF508 mutation.

### Calculations

Each droplet may contain zero copies of a template sequence (negative wells) or one or more copies (positive wells). The true number of copies of each allele in the mixture was calculated by applying a Poisson correction according to the equation: “target counts = -ln [(N-P)/N] x N”, where N is the total number of accepted droplets from the reader, P is the number of positive droplets for the target sequence and ln is the natural logarithm. In addition, the 95% confidence intervals (CI) of true counts of each DNA sequences were calculated as described in Dube et al. [17]. No lower CI was found to lie below zero. A threshold separating negative from positive results was designated at 0.25x the mean value of the positive samples. Fetal fraction was calculated using the formula Pf (%) = ((2*Y) / (X+Y)) *100, where Y is the Poisson-corrected number of ZFY sequences and X is the Poisson-corrected number of ZFX sequences.

### Invasive testing

Fetuses were tested using conventional invasive testing in parallel with the ddPCR assay. Samples were analysed retrospectively but analysts were blinded to the results of the corresponding invasive genetic test performed.

### Ethics

This study was approved by the South Birmingham Research Ethics Committee (REC10/H1207/16; Long-term storage of plasma samples for validation of non-invasive prenatal diagnosis for single gene disorders; Date of favourable ethical opinion: 16th March 2010). Participants provided written informed consent as approved by the ethics committee. All samples were fully anonymised and handled in accordance with the Declaration of Helsinki and ethical approval was granted (REC 10/h1207/16).

## Results

### ΔF508-NOR /ΔF508-MUT quantification

**Fig 2:**
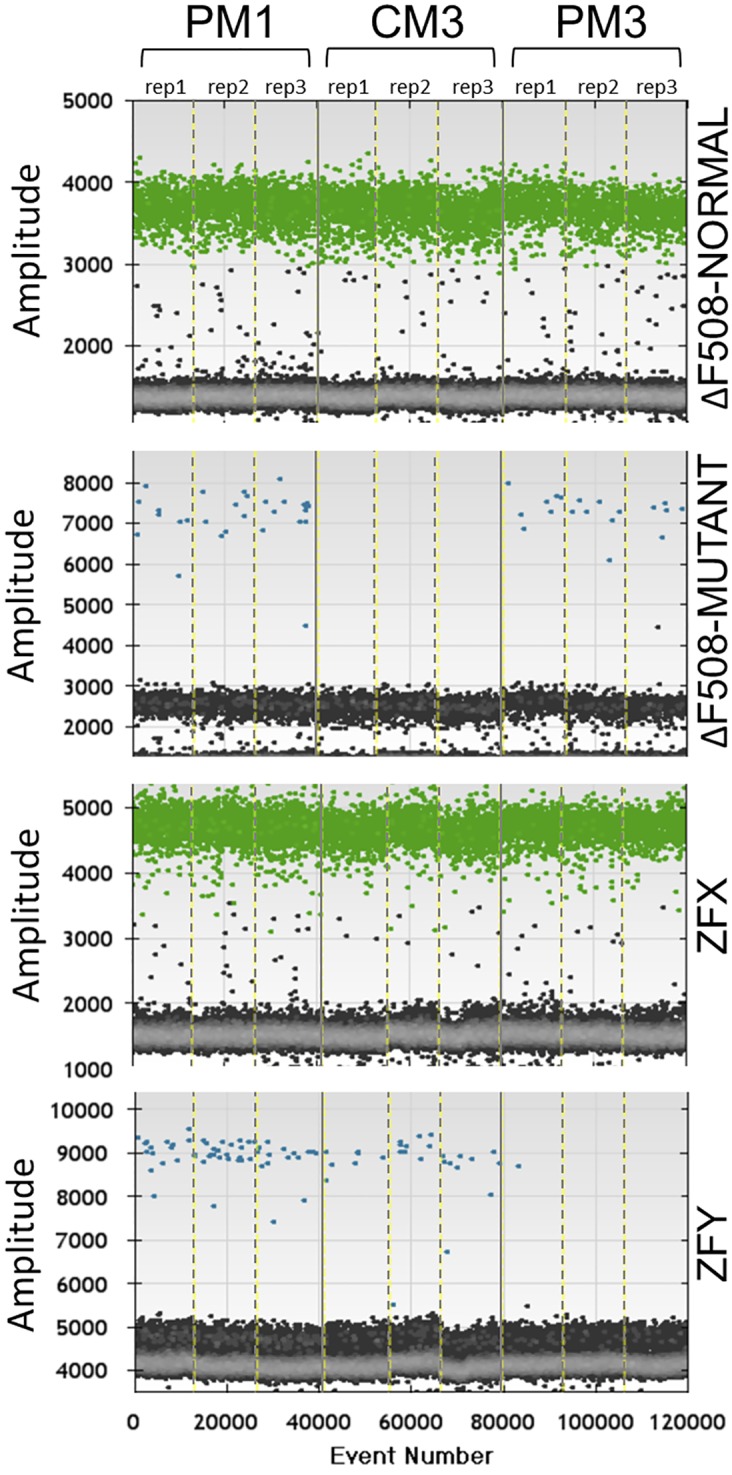
Example ddPCR-NIPD assay results for male proband (PM1), male control (CM3) and female proband (PF3). Three replicates (rep1, rep2 and rep3) are shown for each sample, separated by dashed vertical lines. Each event represents a separate droplet. ZFX and ΔF508-NOR were labelled with VIC and detected at 535–555 nm (positive droplets are shown in green). ZFY and ΔF508-MUT were labelled with FAM and detected at 510–530 nm (positive droplets are shown in blue). Grey points represent negative droplets. As expected, ZFY positive droplets are almost absent in the female sample and ΔF508-MUT positive droplets are absent in the healthy control.

**Fig 3:**
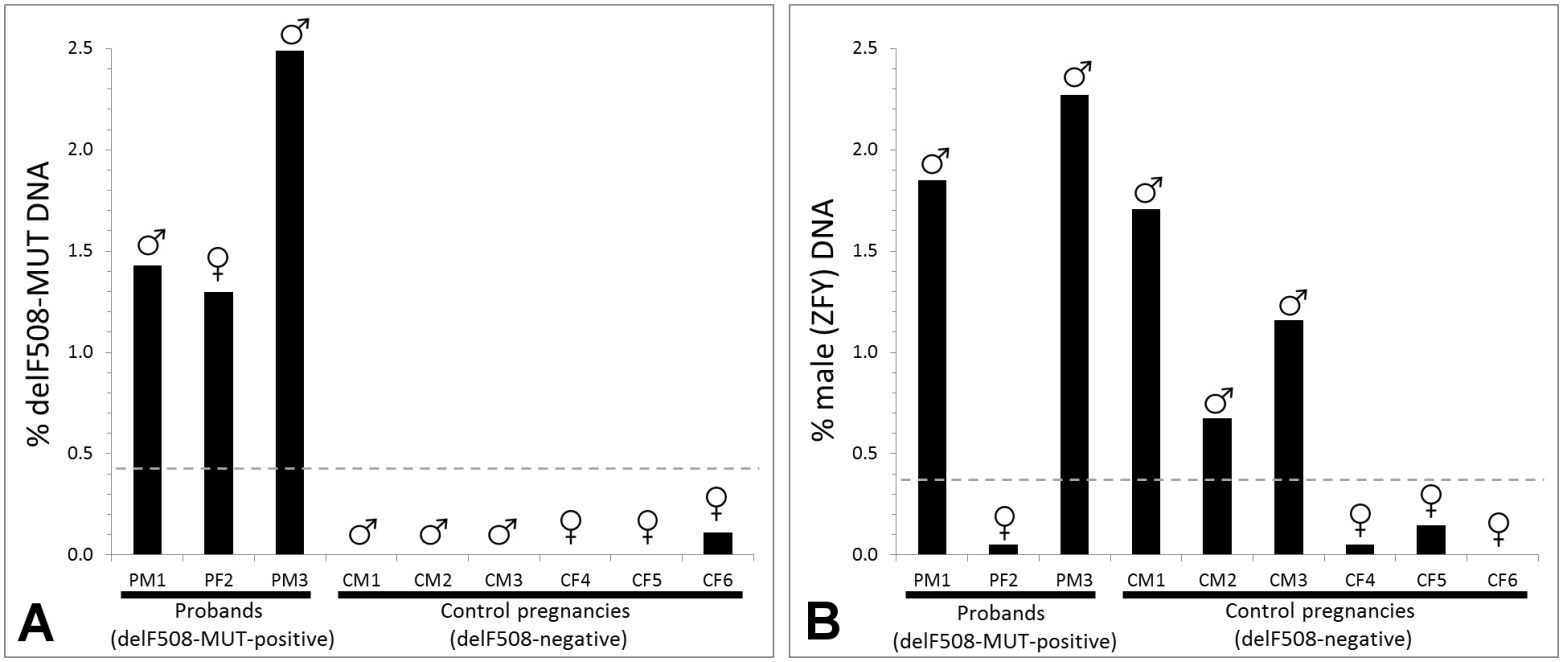
Proportions of ΔF508-MUT (A) and male (B) DNA found in cffDNA. Results are shown for two male probands (PM1, PM3), a female proband (PF2), three control male pregnancies (CM1, CM2, CM3) and three control female pregnancies (CF4, CF5, CF6). Male and female samples are labelled with the (♂) and (♀) symbols respectively. The suggested threshold between a positive and negative result is represented by a grey dashed line at 0.25x the mean of the known positive samples.

The ddPCR assay results were confirmed using the results of a conventional invasive test on the same pregnancies. The ΔF508 mutation was correctly detected (Figs 2 and 3A) in male proband samples PM1 (1.4% of total DNA; 30/2,097 positive droplets) and PM3 (2.4% of total DNA; 37/1,486 positive droplets), as well as the female proband sample PF2 (1.3% of total DNA; 19/1,462 positive droplets). The ΔF508 mutation was not detected in any droplets for five of the six control samples (CM1, CM2, CM3, CF4 and CF5), although a single droplet in sample CF6 was found to be positive for ΔF508. This low level of background (1/892 positive droplets) is very similar to the level seen for the ZFY assays done on female samples PF2 (1/2,010 droplets), CF4 (1/2,049 positive droplets) and CF5 (1/687 positive droplets). These false positives may be caused by droplet carry-over within the instrument during the reading process (Bio-Rad, personal communication).

### cffDNA quantification

The proportions of male DNA estimated by the ZFX/ZFY and ΔF508-NOR /ΔF508-MUT Taqman assays (Fig 3A/3B and S2 Table) were comparable in samples PM1 (3.6% and 2.8% respectively) and PM3 (4.4% and 2.6% respectively). A similar proportion of male DNA was found in the control male samples CM1 (4.4%), CM2 (3.4%) and CM3 (1.3%) using the ZFX/ZFY assay. Female proband sample PF2 was estimated by the ΔF508-NOR /ΔF508-MUT assay to contain 2.4% cffDNA, which is similar to that obtained for the male samples.

### Diagnoses

All three probands in this family were found to carry the paternal ΔF508 mutation, meaning that they each had a 1 in 2 risk of being affected by cystic fibrosis and further (invasive) testing was strongly justified. Conventional invasive testing revealed that probands PM1 and PF2 had also both inherited the mutated maternal allele, while proband PM3 had inherited the normal maternal allele and therefore would be expected to be unaffected by CF.

## Discussion

A Taqman-based ddPCR-cffDNA assay was designed to assess the need for invasive testing in three pregnancies of a couple who were known to carry different *CFTR* alleles. The results of the assay were compared with the results of conventional testing from those three pregnancies as well as three male and three female unrelated control pregnancies.

In a bespoke assay such as this there are insufficient data points to quantify the sensitivity and specificity of the assay as would normally be done for a test designed for a larger population. However, the assay correctly and unambiguously recognized the ΔF508-MUT *CFTR* allele in the cffDNA of all three proband fetuses and none of the six unaffected control fetuses. The level of background observed was low, with 5 of the 6 negative control samples giving a reading of 0% and the final negative control sample giving a reading of 0.1% based on a single positive well (which is less than 8% of the value of the lowest positive result).

There are many options for further optimising this method, for example by running blank samples between experimental samples to prevent carry-over of DNA, by enriching the proportion of cffDNA in the total DNA mixture (e.g. by size fractionation or harvesting at a later trimester), or by increasing the number of droplets or replicates.

Estimates of the fetal fraction vary depending on many factors including fetal age and DNA extraction method. The fetal fractions recovered here (estimated at 2.6–4.9%) were approximately what would be expected for samples taken in the first trimester. The sensitivity of the assay was satisfactory at this level of cffDNA enrichment, in that the assay was easily able to detect mutant alleles making up around 1% of the total DNA mixture. It is probable that the assay would work on mixtures with a somewhat lower Pf (i.e. at a potentially earlier point in gestation) as sensitivities of 0.05–0.1% have been reported [18].

The performance of our assay was at least as good as that of the previously-described ZFX/ZFY assay for detecting male DNA, and this system was overall a cost-effective and practicable testing approach.

## Conclusions

Various high-throughput technologies have been explored to detect paternal cystic fibrosis alleles in cffDNA, such as sequencing [3], microarrays [4] and conventional fluorescence quantitative PCR [5]. We propose that ddPCR is a technically unchallenging, flexible and cost-effective addition to the range of tools available for analysing NIPD samples. It is particularly suitable for applications where accurate quantitation or detection of minor products in a mixture is required as it does not suffer from allele dropout, is absolutely quantitative (unlike microarrays) and can detect as little as 0.05–0.1% [18,19] of a minor product (unlike conventional qPCR which can detect a 2-fold difference). Ultimately the technology used in each NIPD case will depend on the disease being tested and the equipment and expertise possessed by the laboratory.

The assay presented in this study unambiguously recognized the ΔF508-MUT *CFTR* allele in the cffDNA of all three proband fetuses and none of the six unaffected control fetuses. Conventional testing done in parallel confirmed that two of the proband fetuses were compound heterozygotes and that the third would be an unaffected carrier. With some further optimization and validation it could plausibly form the basis for a service aimed at couples whose offspring are known to be at risk of compound heterozygosity for cystic fibrosis.

## Supporting Information

S1 Appendix Further information about the design and completion of the ddPCR assay. (DOCX)

S1 Table Primer and probe sequences and concentrations. (DOCX)

S2 Table ddPCR assay raw data. (DOCX)
