# Supplementary material for: A Non-Invasive Droplet Digital PCR (ddPCR) Assay to Detect Paternal CFTR Mutations in the Cell-Free Fetal DNA (cffDNA) of Three Pregnancies at Risk of Cystic Fibrosis via Compound Heterozygosity
Source: PLoS One. 2015 Nov 11;10(11):e0142729. doi: 10.1371/journal.pone.0142729 (PMC4641687; doi:10.1371/journal.pone.0142729)
Supplement: S1 Appendix — (DOCX) [file pone.0142729.s001.docx]

**S1 appendix.**

Primers were used to co-amplify amplicons of 84bp or 81bp from the wild type and mutant allele respectively (Supplementary table 1). The ddPCR analysis was performed using the QX-100 Bio-Rad system (Bio-Rad laboratories, US), according to the manufacturers’ instructions. All ddPCR reactions were set up using the same cycling conditions, and concentrations of 250nM for each probe and 900nM for each primer, except for Module 1, where primers and probe conditions used are described in section 2.5. PCR reaction for each sample was performed, using 2x ddPCR Supermix for probes (Bio-Rad laboratories, US) in a final reaction volume of 20μl.

Control samples were included in all ddPCR runs. Negative controls were used in order to monitor the occurrence of false positive droplets. Positive controls were used in order to monitor the performance of each assay in all runs and subsequently guide the setting of the threshold between positive and negative partitions for each target sequence. Moreover, in order to eliminate the risk of false-positive results caused by droplet carry-over during the reading process, wells containing the 2x ddPCR Buffer Control solution (Bio-Rad laboratories, US) were added, after a strongly positive control, in some PCR plates.

PCR volumes (20μl) were manually loaded into each of the 8 loading wells of a DG8 droplet generation cartridge and 70μl of droplet generation oil (Bio-Rad laboratories, US) were added to the 8 oil wells. The oil is used in order to produce approximately 20,000 nanoliter droplets for each sample. The samples were carefully loaded to avoid the formation of bubbles, which could interfere with droplet generation. Droplets were generated by the QX100 droplet generator (Bio-Rad laboratories, US) and were manually transferred to a 96-well PCR plate, using an electronic multi-channel pipette. The transfer was done at low and constant speed in order to ensure that the droplets would remain intact prior to amplification. Subsequently, the PCR plate was sealed with foil immediately so as to avoid evaporation.

The thermocycling protocol was: 95°C for 10 minutes, followed by 40 cycles of denaturation at 94°C for 30 seconds and annealing/extension at 57°C for 1 minute, with a slow ramp of 2,5°C per second, followed by a step at 98°C for 10 minutes and infinite hold at 12°C.

After PCR amplification, the reactions were automatically analyzed on the QX100 droplet reader (Bio-Rad laboratories), which is a fluorescence detector, while the data were processed by the QuantaSoft™ PCR analysis software (Bio-Rad laboratories, US). Additionally, the computer analysis that was done in the following section was performed using the Microsoft Office Excel software.

PCR efficiency was found to be optimal at 56.7°C for the ZFX/ZFY assay, but 56°C for the delF508 assay. In order to run all three assays on the same PCR plate, a compromise temperature of 57°C was used. The assays were found to perform best with 250 nM of each probe for both assays. These optimized conditions showed the best separation between positive, negative and cross-hybridized partitions (not shown).
