# Supplementary material for: A Non-Invasive Droplet Digital PCR (ddPCR) Assay to Detect Paternal CFTR Mutations in the Cell-Free Fetal DNA (cffDNA) of Three Pregnancies at Risk of Cystic Fibrosis via Compound Heterozygosity
Source: PLoS One. 2015 Nov 11;10(11):e0142729. doi: 10.1371/journal.pone.0142729 (PMC4641687; doi:10.1371/journal.pone.0142729)
Supplement: S1 Table — (DOCX) [file pone.0142729.s002.docx]

**S1 table.**

| **Target** |  | **Sequence** | **Final concentration (nM)** |
| --- | --- | --- | --- |
| *ZFX/ZFY ^a^* | ZF2 primer | 5'-CAAGTGCTGGACTCAGATGTAACT-3' | 900 |
|  | ZR2-primer | 5'-TGAAGTAATGTCAGAAGCTAAAACATC-3' | 900 |
|  | Y-probe | 5'-(FAM)TCTTTACCACACTGCAC(MGBNFQ)-3’ ^b^ | 250 |
|  | X-probe | 5'-(VIC)TCTTTAGCACATTGCA(MGBNFQ)-3' ^b^ | 250 |
| *CFTR* deltaF508 ^c^ | For primer | 5'-CTCAGTTTTCCTGGATTATG-3' | 900 |
|  | Rev primer | 5'-CGCTTCTGTATCTATATTCATC-3' | 900 |
|  | MUT probe | 5'-(FAM)ATATCATTGGTGTTTCC(MGBNFQ)-3' | 250 |
|  | NOR probe | 5'-(VIC)ATATCATCTTTGGTGTTTC(MGBNFQ)-3' | 250 |
|  |  |  |  |
|  |  |  |  |
| ^a^ VIC and FAM, fluorescent reporter dyes; MGBNFQ, minor groove binding non-fluorescent quencher; nucleotide differences between allelic probes are underlined. | | | |
| ^b^ as described in ref. 2 with the exception of primer ZR2. | | |  |
| ^c^ MUT: delF508 mutation probe; NOR: normal allele probe. | | |  |
|  |  |  |  |
