# Supplementary material for: A Non-Invasive Droplet Digital PCR (ddPCR) Assay to Detect Paternal CFTR Mutations in the Cell-Free Fetal DNA (cffDNA) of Three Pregnancies at Risk of Cystic Fibrosis via Compound Heterozygosity
Source: PLoS One. 2015 Nov 11;10(11):e0142729. doi: 10.1371/journal.pone.0142729 (PMC4641687; doi:10.1371/journal.pone.0142729)
Supplement: S2 Table — (DOCX) [file pone.0142729.s003.docx]

**S2 table.**
